# ANDES: Statistical tools for the ANalyses of DEep Sequencing

**DOI:** 10.1186/1756-0500-3-199

**Published:** 2010-07-15

**Authors:** Kelvin Li, Eli Venter, Shibu Yooseph, Timothy B Stockwell, Lance D Eckerle, Mark R Denison, David J Spiro, Barbara A Methé

**Affiliations:** 1 The J. Craig Venter Institute, 9704 Medical Center Drive, Rockville, MD 20850, USA; 2 Vanderbilt University Medical Center, D6217 MCN, Nashville, TN 37232-2581, USA

## Abstract

**Background:**

The advancements in DNA sequencing technologies have allowed researchers to progress from the analyses of a single organism towards the deep sequencing of a sample of organisms. With sufficient sequencing depth, it is now possible to detect subtle variations between members of the same species, or between mixed species with shared biomarkers, such as the 16S rRNA gene. However, traditional sequencing analyses of samples from largely homogeneous populations are often still based on multiple sequence alignments (MSA), where each sequence is placed along a separate row and similarities between aligned bases can be followed down each column. While this visual format is intuitive for a small set of aligned sequences, the representation quickly becomes cumbersome as sequencing depths cover loci hundreds or thousands of reads deep.

**Findings:**

We have developed ANDES, a software library and a suite of applications, written in Perl and R, for the statistical ANalyses of DEep Sequencing. The fundamental data structure underlying ANDES is the position profile, which contains the nucleotide distributions for each genomic position resultant from a multiple sequence alignment (MSA). Tools include the root mean square deviation (RMSD) plot, which allows for the visual comparison of multiple samples on a position-by-position basis, and the computation of base conversion frequencies (transition/transversion rates), variation (Shannon entropy), inter-sample clustering and visualization (dendrogram and multidimensional scaling (MDS) plot), threshold-driven consensus sequence generation and polymorphism detection, and the estimation of empirically determined sequencing quality values.

**Conclusions:**

As new sequencing technologies evolve, deep sequencing will become increasingly cost-efficient and the inter and intra-sample comparisons of largely homogeneous sequences will become more common. We have provided a software package and demonstrated its application on various empirically-derived datasets. Investigators may download the software from Sourceforge at https://sourceforge.net/projects/andestools.

## Findings

The advancements in next generation sequencing technologies, such as 454 [1] or Solexa [2], have led to an increased interest in the deep sequencing of samples from mixed populations. When the sequences of interest are largely similar and only a few salient differences exist within these populations, it is difficult to quantify or to identify the important trends with existing tools. The MSA is a useful visual aid when analyzing a small set of sequences within a sample, but to identify important trends within a large number of sequences, or among multiple sets of deeply sequenced samples, a more quantitative approach is necessary. Existing tools, such as BioPerl [3], BioPython [4], and SAMtools [5], are useful for efficiently generating, manipulating and storing MSAs, but statistical metrics and visualizations for the comparison of the samples that the MSAs represent, do not exist.

The applications we have implemented in ANDES allow an investigator to identify and compare the variations within a sample or among multiple samples. Three independent projects that have used ANDES will be described in the "Results and Discussion" section. The first project involved quantifying, on a whole genome level, the mutation rates and the locations of novel variations for a coronavirus that had been passaged 10 consecutive times in cell culture. The depth of sequencing was derived from a single Solexa run for each of 3 selected passages. This exemplifies how ANDES can be used to compare temporally separated samples, to analyze genome-wide mutation trends, and to identify specific variations for further analyses. In the second project, ANDES was used to differentiate between the strains of human influenza H1N1 sequences that were deposited into GenBank. Because the majority of human H1N1 sequences were generated from the circulating seasonal strain, there were little, or no, consistent annotations in the GenBank records to differentiate them from the collected sequences of the 2009 swine-origin influenza pandemic. In this case, the sequencing depth was accumulated across data that were deposited by a community of influenza researchers. The clustering and analyses of the dataset made it possible to design strain-optimized, degenerate PCR primers for the purpose of sequencing both seasonal and swine origin H1N1 genomes. The final project described is an application of the ANDES toolset to assess the sequencing accuracy and identify loci-specific sequencing errors for a 454 Titanium sequencing run performed on a subregion of the 16S rRNA gene (16S rDNA). The deep sequencing of clonal copies of a 16S sequence provided the data to empirically determine actual PCR and sequencing error rates. This has played a critical role in characterizing the nature of sequencing errors on downstream analyses.

## Implementation

### Position profile

**Figure 1:**
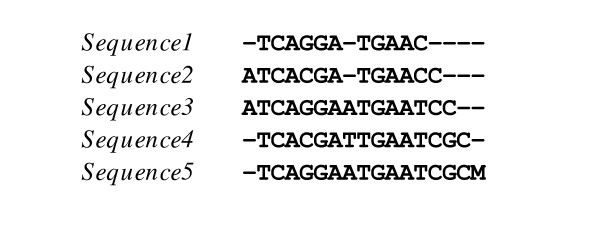
**Example multiple sequence alignment (MSA)**.

**Figure 2:**
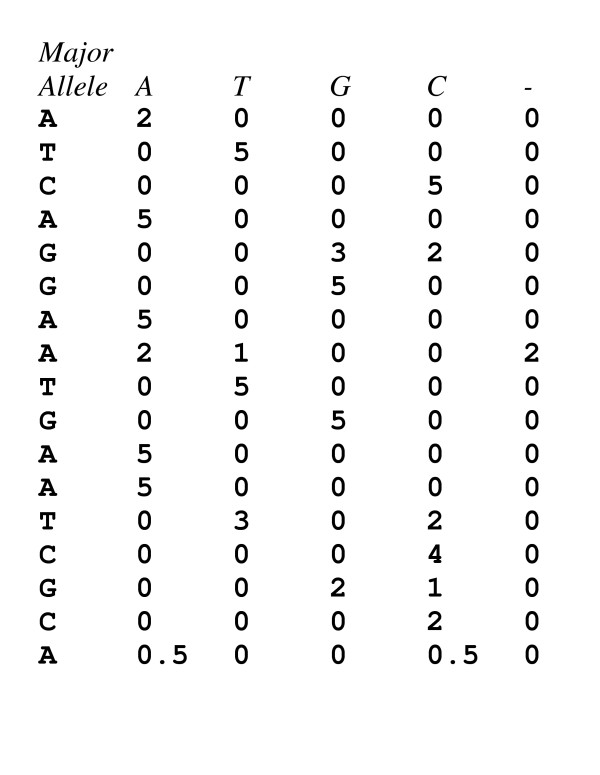
**Example position profile for the MSA shown in Figure 1**. Column 1 contains the most common allele. When multiple alleles share the value of the greatest frequency, one of the alleles is arbitrarily chosen to represent that position. Columns 2-5 represent the frequency of each nucleotide at each position for the sample represented by the position profile.

The fundamental data structure of the ANDES suite of tools is the *position profile*. This data structure contains the number of nucleotides that support each position of the analyzed region of interest. ANDES currently supports the conversion from the output of clustalw[6] (*i.e*. .aln files), however the format of the position profile is relatively trivial; therefore the encoding of output from an alternative alignment application should be readily supported. Alignments generated with RazerS[7], MUSCLE [8], and AMOScmp [9] tools have also been successfully converted into position profiles and analyzed. Figure 1 shows the input MSA that the resultant position profile in Figure 2 was created from. Each column in the position profile is calculated by summing the number of nucleotides or gap contributions at each position. Each line of the position profile is essentially a probability mass function (pmf), with support at {A, T, G, C, and - (gap)}, when the values are normalized and sum to 1. Gaps introduced at the beginning or end of the MSA are assumed to be caused by read termination, so these gaps are not considered to be nucleotide variations and are therefore ignored. Bases with IUPAC ambiguity codes [10] in the alignment are given equal weight for each nucleotide they represent. For example, the IUPAC code, M, represents A or C, so 0.5 will be contributed to both A and C position counts. The major allele is also stored in the profile for visual convenience. The number of lines in the position profile is the gapped length of the consensus sequence for the MSA.

### Aligning profiles for comparison and merging

The alignment of two or more profiles is important for comparing or merging their contents into a new composite profile. Profile merging may be used to pool multiple sample profiles together, or to accumulate the sequencing results from a previously partitioned sample. Since the compute time of an all-versus-all MSA tool, such as clustalw, has an exponential growth rate [8] as a function of the number of sequences in a run, *N*, it is recommended to reduce *N*, and take advantage of parallel computation resources. This can be accomplished by dividing *N *across *S *independent runs with smaller input sizes of *N/S*, and then performing a final merge across the *S *profiles.

Profile alignment in ANDES involves computing the major allele for each position based on the underlying nucleotide composition. If the most common allele at a position is a gap, then the most frequent allele that caused the gap to open is used as the major allele. The representative major alleles for each profile are then aligned with clustalw. The results from the clustalw alignment are then used as a proxy to the underlying nucleotide distributions. Since the quality of the profile alignment can only be as good as the algorithms used to align the major alleles, ANDES also allows for the integration of alternative MSA tools, such as MUSCLE, which can also produce clustalw-like output. To complete the profile merge, the new position frequencies are then summed for each column, using the major allele positions as proxies. Similarly for a profile comparison, the corresponding positional frequencies between the two aligned profiles are then compared.

### Root mean square deviation plot

**Figure 3:**
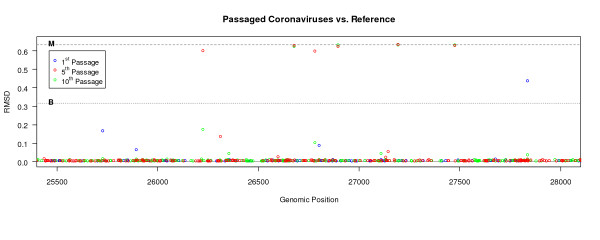
**Example RMSD plot**. This plot compares 3 Solexa sequenced samples against a Sanger sequenced reference. The blue, green, and red glyphs represent samples from the 1^st^, 5^th ^and 10^th ^passages, respectively, of the coronavirus. The genomic position is on the x-axis, and the y-axis represents the RMSD distance from each sample to the reference. The M and B annotated dashed lines represent the maximum and biallelic RMSD levels, 0.632 and 0.316 respectively, explained in Figures 4 and 5.

The root mean square deviation (RMSD) plot is a visualization of the differences between the nucleotide distributions along the alignment of two or more samples. In the RMSD plot, the alignment between two sequences is represented along the x-axis, and the computed RMSD value for each nucleotide position between two aligned samples is represented along the y-axis (Figure 3). To compute the RMSD for each nucleotide position, the standard RMSD formula [11] was modified to compare the nucleotide distributions between each sample:

$$RMSD{\left(X,Y\right)}_{i}=\sqrt{\frac{\Sigma_{p=zA,T,G,C,-\}}{\left(P[X_{i,p}]-P[Y_{i,p}]\right)}^{2}}{5}}$$

*P[X*_*i,p*_] and *P[Y*_*i,p*_] are the probabilities of nucleotide, *p*, at position *i*, for the sample X and Y, respectively. The nucleotide, *p*, is an element of the nucleotide set {A, T, G, C, -}, where the - character, represents a gap that was necessary to align the two profiles. The position, *i*, ranges from 1 to the length of the gapped alignment, *L*. The number of insertions and deletions (indels) that were necessary to align the sequences in the MSA, are also included for each position. The denominator within the square root operator, 5, is the number of symbols in the nucleotide set used.

**Figure 4:**
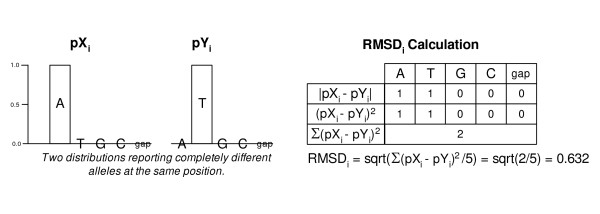
**Example calculation of the RMSD value between two monoallelic samples, X and Y**. In this case, the maximum RMSD value of 0.632 was achieved because the allelic distributions between the two sample positions did not agree.

**Figure 5:**
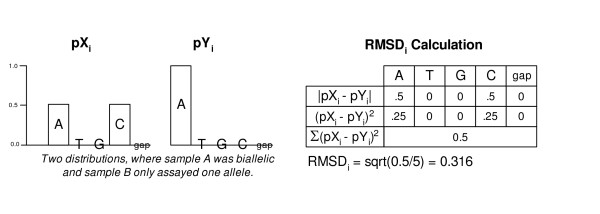
**Example calculation of the RMSD value between a biallelic sample, X, and a monoallelic sample, Y**. In this case, the intermediate RMSD value of 0.316 was generated because sample X was biallelic, and sample Y was monoallelic but in partial agreement.

The minimum RMSD value of 0 occurs when the distributions of nucleotides are identical between the two alignments for a specific position. The maximum value of 0.632 is generated if the allele of one sample is completely different than the second sample, for example, if sample X consisted of 100% T's and sample Y consisted of 100% G's. Another important RMSD value is 0.316. This value occurs when one sample is biallelic, for example A/T, and the other sample is partially similar with a single allelic representation of A. See Figure 4 and 5 for sample calculations of the two cases.

Summary RMSD values between two samples can be computed by taking the arithmetic mean of the RMSD value for every position along the length of the alignment, *L*. These summary values can be used to generate a distance matrix among all samples. See Table 1.

**Table 1:** Example distance matrix table.

	1-Jun-08	16-Jun-08	16-Nov-08	1-Dec-08	16-Dec-08
**1-Jun-08**	0.00000	0.01297	0.03203	0.03184	0.02221
**16-Jun-08**	0.01297	0.00000	0.02209	0.02191	0.01220
**16-Nov-08**	0.03203	0.02209	0.00000	0.00247	0.01054
**1-Dec-08**	0.03184	0.02191	0.00247	0.00000	0.01043
**16-Dec-08**	0.02221	0.01220	0.01054	0.01043	0.00000

This table represents a subset of the RMSD-based distances that were calculated between partitions of human influenza H1N1 collection dates. Each value is the average positional RMSD along the lengths of the 2 compared HA segments. The complete RMSD distance matrix was then used as input into R to compute a dendrogram as seen in Figure 7.

### Shannon entropy plot

The Shannon entropy (SE) is a measure of uncertainty or information content in a random variable. When applied to the distribution of nucleotides at a single position, it represents the amount of variation at that position. A normalized Shannon entropy (NSE) can be computed by using a logarithm of base 5, the number of supports for the distribution, for the calculation. This NSE has a range between 0 and 1, where 0 represents no variation (only a single allele is represented in the sample), and 1 represents maximum variation (the uniform existence of A, T, G, C and gaps).

$$SE{\left(X\right)}_{i}=-\sum_{p=zA,T,G,C,-\}}P[X_{i,p}]\log_{5}(P[X_{i,p}])$$

**Figure 6:**
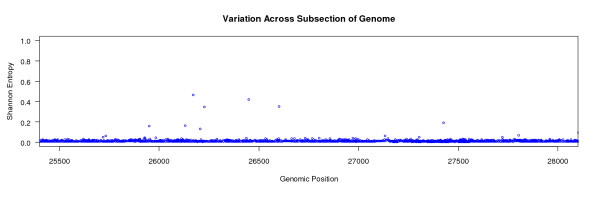
**Example Shannon entropy plot**. This plot shows the variation, measured by the Shannon entropy, for the 10^th ^passage of the coronavirus. The x-axis is the genomic position, and the y-axis is the Shannon entropy.

*P[X_i,p_] *is the probability of nucleotide, *p*, at position *i*, for the sample X. The nucleotide, *p*, is an element of {A, T, G, C, -}, where the - character, represents a gap that was necessary to align all the sequences within the sample. The position, *i*, ranges from 1 to the length of the gapped alignment, *L*. The number of insertions and deletions (indels) which were necessary to align the sequences in the MSA, are also included for each position. The SE plot is generated by plotting the alignment of a single sample along the x-axis versus the SE on the y-axis (for example, Figure 6). A summary SE value for a single sample can be computed by taking the arithmetic mean of the SE for every position along the length of the alignment, *L*. Since the SE can be calculated within a single sample, it is useful for quickly identifying regions or positions of variability without the necessity of choosing an ideal reference sequence.

### Nucleotide conversion analyses

The base conversion probabilities between two samples, or within a single sample, can be computed between aligned profiles. Given an alignment of all sequences in the sample(s), the following formula, which is based on the definition of conditional probability, is utilized:

$$P[X_{p}|Y_{q}]=\frac{\sum_{i=1}^{L}P[X_{i,p}]P[Y_{i,q}]}{\sum_{i=1}^{L}P[Y_{i,q}]}$$

This formula computes the probability of base *q *in aligned position *i *in Sample Y, converting to base *p *in aligned position *i *in Sample X, summed over all aligned positions. *p *and *q *are from the nucleotide set {A, T, G, C, -}. *i *is the nucleotide position range between 1 to the length of the gapped alignment, *L*. Computing base conversion frequencies is useful for determining if there are any unexpected pressures in mutation rates towards particular nucleotides, or sequencing error biases. One will typically see a significantly higher base conversion rate between transitions (A↔G or C↔T) than transversions (other remaining combinations).

### Inter-sample cluster analyses

Inter-sample cluster analyses can be performed by first computing the distance matrix among all samples with the RMSD formula.

$$Distance(X,Y)=\frac{1}{L}\sum_{i=1}^{L}RMSD{\left(X,Y\right)}_{i}$$

The matrix can be loaded into a statistics package such as R [12], and a multidimensional scaling (MDS) plot or dendrogram may be computed and plotted. MDS is a statistical technique used to convert an N dimensional description of the relationship between samples (e.g., a distance matrix), into a lower dimensional plot (e.g., 2D). As a result, samples with similar characteristics are reflected in the plot by their close spatial proximity to each other. Sample R code has been provided in the ANDES tool set to perform these cluster analyses based on the generated distance matrices.

### Threshold-driven consensus generation and polymorphism detection

The detection of polymorphisms and the generation of a representative consensus sequence both depend on the investigators' threshold for determining the significance of underlying variations. For example, this threshold may be the rate of sequencing errors or the prevalence of a minor allele that the investigator would like to eliminate from the consensus sequence. It is common to automatically assign IUPAC ambiguity codes to certain base positions even when the existence of the alternative base, or bases, has a low frequency, e.g., 1 representative. Alternatively, consensus sequences may be manually edited or entire sequences may be eliminated from the set of representative input sequences, in the effort to reduce the dominance of ambiguity codes in the final consensus sequence. These workarounds are tedious, and the elimination of specific sequences from the set of sequences under analysis leaves the final consensus sequence potentially skewed towards the representation of only the most common variants. Downstream processes, such as degenerate primer design will then be forced to compute on a non-representative consensus. To generate the most representative consensus sequence, it is preferable to use the largest sample possible, since this will represent the population of interest most accurately and minimize the effects of random errors.

Profile to consensus generation automatically converts a position profile into a consensus sequence using the standard IUPAC ambiguity codes. The benefit of using a position profile to generate a consensus sequence is the ability to perform filtering *a priori*, using either a nominal or percent cutoff. When a nominal threshold-based filter is applied, the nucleotide frequencies for each position that do not exceed the specified threshold are set to zero. By not changing the values of the nucleotide frequencies that exceed the threshold, their relative proportions are still maintained. When applying a percent cutoff filter, the positional nucleotide frequencies are first normalized into positional probability mass functions (pmf's). An application of threshold-based filtering can be to remove low levels of nucleotide representations as a result of sequencing errors, or to prioritize the depth of variation allowed in the consensus sequence.

**Figure 7:**
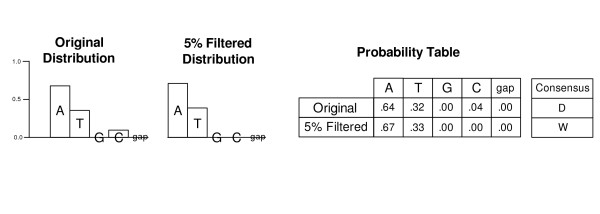
**Example effect of percentage filtering used to reduce the coverage of ambiguity codes**. In the original nucleotide distribution for a specific position, the ambiguity code D, which represents A, G, or T is called. C is likely to be a result of sequencing error, so it is preferable to exclude it when designing degenerate primers. After applying a 5% filter, the assigned ambiguity code for that position can be reduced to a W, which more accurately represents the target population (A or T).

An example of the effect of filtering based on nucleotide probability is shown in Figure 7. In this example, the ambiguity code assigned to the position was D, because the position required representation for the existence of A, T, and C in the sample. A sequencing error upper bound rate of 5% was determined and used as a threshold for filtering low prevalence nucleotide representations. The necessity for a C representation was removed, allowing the new ambiguity code to become a W, representing A or T. Assuming the representation of C was a result of sequencing error, the W ambiguity code is a more accurate representation of the allelic composition at that position for the sample.

### Generation of empirically characterized quality values

If deep sequencing is performed on a homogenous or clonal population, then it is possible to estimate the average quality value (QV) that should have been assigned to each position independent of the quality values assigned by the sequencing technology's base caller. This is useful for comparing the actual error rate to that of the base caller's quality value, for example to properly calibrate the sequencing QV or to set alternative thresholds of sequence quality based on actual acceptable error rates. The standard formula for QV is:

$$QV_{i}=-10\;log_{10}(P[Err_{i}])$$

The probability of an error, *P[Err*_*i*_], is estimated for each position *i*, as:

$$P[Err_{i}]=1-\left\{\max_{p=zA,T,G,C,-\}}P[X_{i,p}]\right\}$$

The probability of an error is defined as the probability of not attaining the most common allele. If the empirically determined error rate is 0, then the QV is set to 40 (i.e., 0.0001 errors per base). If the coverage depth allows for the determination of error rates less than 0.0001, then a QV of greater than 40 is optionally permitted. Most base callers limit their maximum QV to 40, although there is no theoretical limit.

## Results and discussion

### Profile Alignment

To compare or merge the positional nucleotide distributions across multiple profiles, it is necessary to first perform a global profile alignment, so that homologous nucleotide positions can be determined. To demonstrate the profile alignment strategy based on using the major allele as a proxy, 2,900 sequences (a sample size divisible by 2, 4, and 10) were randomly sampled from the 2,913 Avian influenza MP segments that were available from Genbank and multiple degrees of partitioning and merging were compared against the reference profile, which was generated without any partitioning or merging. The results of this experiment show the effect of partition size and count, on both profile generation accuracy and compute time.

**Figure 8:**
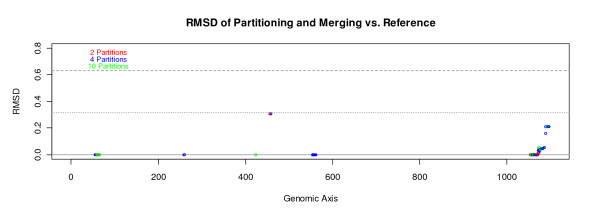
**RMSD plot comparing the partitioning and merging of 2900 avian influenza MP segments against the reference unpartitioned/unmerged profile**. The majority of positions are identical to the reference. Exceptions occur around 450 bp, where a tandem repeat causes a MSA error, and at the 3' end of the profile, where even the reference alignment is questionable due to poor sequencing quality.

The accuracy of the partition and merge strategy was measured and illustrated with the RMSD plot. The split and merge of 2, 4, and 10 partitions, were plotted against the reference as seen in Figure 8. Most nucleotide distributions between merged profiles were identical to the reference with an RMSD value of 0. Exceptions occurred around 450 bp (a dinucleotide repeat) and at the 5' and 3' ends, where even the reference MSA was questionable due to improper gapping. The combination of lower sequencing quality towards the 3' end of sequences, mismatching sequence lengths, and short tandem repeats, typically contribute to poor end MSAs. Overall, the merging of profiles was accurate even across 10 partitions.

Timing statistics measuring the reduction of both serial and parallel compute times using this partition and merge strategy are laid out in Table 2. Parallel compute times were estimated as the sum of the parallel times for sequence alignment, profile generation, and profile merge. The serial compute times used the sum of the serial times for the same 3 steps. The parallel compute time for each step was estimated as the longest running job among partition compute times, and the serial compute time was estimated as the sum of all partition compute times. When the input sequences were split into 10 partitions, serial and parallel speedup factors of 4.4x and 41.35x were observed, respectively, with very little degradation of profile generation accuracy.

**Table 2:** Time (user + system) in seconds for generating profiles with various partitions sizes on 2900 avian MP segments

Number of Partitions	1	2	4	10
Number of Sequences/Partition	2900	1450	725	290
**Sequence Alignment Times:**	3179.73	882.88	282.43	74.37
(with clustalw2 -quicktree)		896.14	284.10	70.70
			275.90	70.83
			274.19	70.46
				71.59
				71.06
				70.69
				71.02
				71.28
				71.04
	
*Parallel Time (max)*	*3179.73*	*896.14*	*284.10*	*74.37*
*Serial Time (sum)*	*3179.73*	*1779.02*	*1116.62*	*713.04*

**Profile Generation Times:**	11.50	7.61	2.76	1.11
		6.15	2.74	1.17
			2.93	1.09
			2.86	1.14
				1.13
				1.12
				1.16
				1.10
				1.17
				1.24
	
*Parallel Time (max)*	*11.50*	*7.61*	*2.93*	*1.24*
*Serial Time (sum)*	*11.50*	*13.76*	*11.29*	*11.43*

**Profile Merge Times:**	0.00	0.30	0.63	1.57
	
*Parallel Time (max)*	*0.00*	*0.30*	*0.63*	*1.57*
*Serial Time (sum)*	*0.00*	*0.30*	*0.63*	*1.57*

**Total Parallel Times**	***3191.23***	***904.05***	***287.66***	***77.18***
**Total Serial Times**	***3191.23***	***1793.08***	***1128.54***	***726.04***
**Parallel Speed Up Factor**	**1.00**	**3.53**	**11.09**	**41.35**
**Serial Speed Up Factor**	**1.00**	**1.78**	**2.83**	**4.40**

2900 avian MP segments were randomly selected without replacement from the available 2913 segments in Genbank. The sequences were then partitioned into 2, 4, and 10 subsets to demonstrate the effect of partitioning on profile generation time and accuracy. Individual compute times for sequence alignment, profile generation, and profile merge were recorded for each job. Parallel and serial compute times for sequence alignment and profile generation were computed by finding the maximum and sum, respectively, across individual job times. Total parallel and serial compute times for each partition were calculated by summing up the parallel and serial compute times, respectively, across sequence alignment, profile generation, and profile merge. When partitioned into 10 subsets, serial and parallel speedup factors of 4.4 and 41.35 were observed, respectively.

### Root mean square deviation plot

Deep sequencing platforms such as 454, Solexa and SOLiD provide opportunities to explore genetic diversity of populations at much greater resolution than Sanger sequencing, Development of quantitative and visual analyses of genome-wide variations are critical for identifying trends and assessing their biological significance. Figure 3 is an example of a root mean square deviation (RMSD) plot applied to a study of viral diversity using deep sequencing data that was generated from a series of coronavirus (CoV) passages. The coronavirus utilized in this study was engineered to produce a rapidly mutating phenotype. Viral RNA from the 1^st^, 5^th ^and 10^th ^passages was subjected to RT-PCR and amplicons were deeply sequenced with Solexa [13]. Reads were mapped onto a Sanger sequenced reference CoV sequence, GenBank accession AY278741, using RazerS, and a position profile was generated for each of the sequenced passages. The approximate coverage depth for base positions ranged from 500x to 1000x. There was significantly greater sequencing depth for positions covered by overlapping amplicons. The RMSD plot was then generated based on the positional RMSD values computed between the position profiles for each of the passages and the reference. The blue, green, and red glyphs represent variations from the 1^st^, 5^th^, and 10^th ^passages, respectively, for each base position. From the RMSD plot, one can determine that the positional distributions of nucleotides have increasingly deviated from the reference. With the association of annotation (not presented), an investigator can quickly determine which genes may have been impacted and focus on their analyses.

### Shannon entropy plot

The Shannon entropy (SE) plot was then generated to further investigate the variation within the sample of the CoV population sequenced in the 10^th ^passage (Figure 6). The SE plot facilitates distinguishing which positions in the sequenced genomes have higher than background levels of variation. Most of the positions in the analyzed region from 25,500 - 28,000 bp, have an SE close to zero, which would indicate that a single allele was dominant. An SE analysis over time may show SE values for specific positions increase as the viral population began to adapt to growth in the cell culture, and then decrease if an advantageous variation increasingly dominates in the population. Positions with consistently high SE values over time may be experiencing little or no selective pressure. For example, in Figure 6, the variation seen in the SE plot was higher than the background in the region between 26,000 and 26,700. This region encompasses the envelope protein and membrane protein ORFs of the CoV. This data may suggest to researchers that an experiment could be designed to investigate the role of these particular proteins to the CoV's adaptation to growth in cell culture.

### Nucleotide conversion analysis table

A nucleotide conversion table was computed between the 1^st ^and 10^th ^passage on the same region of the CoV genome that was analyzed for the RMSD and SE plots (Table 3). This nucleotide conversion table summarizes the probability that a base in the 1^st ^passage will change to another base by the 10^th ^passage. 1^st ^passage nucleotides have been labeled for each row on the left of the table and 10^th ^passage nucleotides have been labeled for each column at the top of the table. For example, there is a 0.47% probability that a G in the 1^st ^passage, will become an A by the 10^th ^passage. Transition probabilities (A↔G or C↔T), which have been highlighted in bold, tend to be greater, than transversion probabilities. From the table, the probability of the transition from G→A (0.47%) is greater than the transversion probabilities for G→T (0.08%) or G→C (0.06%). However, the A→T (0.24%) and T→G (0.35%) transversions have higher rates than the A→G (0.18%) and T→C (0.28%) transitions. This contributes additional evidence that there may be some selective pressure acting upon this region of the genome, especially if these probabilities depart significantly from those computed for the entire length of the genome when using the same sequencing technology.

**Table 3:** Example nucleotide conversion analysis table

		*10th Passage*				
		A	T	G	C	-
***1st Passage***	**A**	98.99%	0.24%	**0.18%**	0.12%	0.47%
	**T**	0.20%	98.78%	0.35%	**0.28%**	0.40%
	**G**	**0.47%**	0.08%	99.18%	0.06%	0.22%
	**C**	0.10%	**0.67%**	0.05%	98.97%	0.21%
	**-**	13.74%	33.03%	22.68%	30.35%	0.19%

The source nucleotide is represented along the rows. The destination nucleotide is represented along the columns. To interpret the measure at 1^st ^passage G/10^th ^passage A, one could say there is a 0.47% probability that a G in the first passage will become an A at the 10^th ^passage, P(A in 10^th ^passage ∣ G in 1^st^ passage) = 0.47%. Transition frequencies have been highlighted in bold. Because the table contains conditional probabilities, i.e. not marginal probabilities, the percentages in each row will sum to 100%, but the percentages down each column may not.

Establishing a baseline for sequencing technology specific systematic sequencing errors is especially important for the nucleotide conversion analyses, since these computed values are absolute, rather than relative to another sample's positional nucleotide distribution. Since every read contributes to the overall analysis, rather than a vote towards a consensus sequence, the impact of sequencing errors on individual reads must be considered. Most sequencing technologies either include a step to amplify the target DNA or require quantities of DNA which required the investigator to perform DNA amplification *a priori*. It has been estimated that PCR amplification will introduce an upper bound on sequence quality which is dependent on the fidelity of the polymerase. Excluding PCR slippage and mispriming, Taq DNA polymerase has been demonstrated to produce error rates of at least 10^-5 ^for base substitutions and 10^-6 ^for frameshift errors [14]. The application of the nucleotide distribution-based filtering tools, an integral part of ANDES, allows the user to define a threshold for differentiating between base conversions which may be significant, versus those that should be attributed to sequencing or PCR errors.

### RMSD-based distance matrix and dendrogram

**Figure 9:**
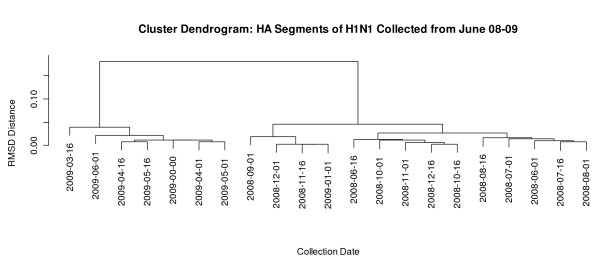
**Example dendrogram clustering using an RMSD-based distance matrix**. Each leaf in the dendrogram represents a biweekly partition of collection dates. The cluster of leaves on the left-hand side are likely attributed to the swine origin H1N1 virus outbreak, whereas those sequences collected on the right-hand side were based on the circulating seasonal H1N1 virus.

An important capability of ANDES is the ability to compute the summary distances between deep sequencing results to produce an N × N distance matrix. When a large group of sequences can be partitioned, for example temporally, ANDES can be used to quantify the differences among each partition. With the help of statistical cluster analyses tools, partitions that contain the variations of interest can be identified. As an example, the hemagglutinin (HA) coding segments of human influenza A H1N1 sequences were downloaded from NCBI and were grouped according to the collection date that was annotated in their respective GenBank records. Sequences were grouped into biweekly partitions when possible. If the collection day was not available, then the sequence was assigned to the partition with the same month with a null day. For example, if the collection date for a sample was June 2008, then the sample's sequence would be assigned to the 2008-06-00 partition. If the collection year was the only information recorded, then the sequence would be assigned to a partition with a null month and a null day, for example 2008-00-00. For each partition, clustalw was used to align all the sequences within the partition and a position profile was generated. A distance matrix was then generated by computing the summary RMSD value between every position profile, and R, the statistical software package, was used to produce a dendrogram with the hclust function (Figure 9). As apparent from the dendrogram, the human H1N1 samples that were collected from March to June 2009 have much greater sequence similarity than those collected from June 2008 to January 2009. This may be attributed to the sudden focus of H1N1 sequencing performed on potential swine origin viral samples that were collected starting March 2009. Many other applications can be imagined for comparing position profiles across partitions, for example, investigating influenza diversity among different avian species or association of viral clades by geographic location.

### Threshold-driven consensus generation

The ability to analyze groups of similar sequences that have been objectively partitioned either spatially or temporally allows individual partitions of interest to be identified. The underlying position profiles of these partitions can then be merged together to generate a new cumulative position profile from which a consensus sequence can be generated for downstream applications, such as degenerate primer design. For example, suppose it was determined that the mutation rate for a circulating virus caused a percentage of previously designed primers to fail at an unacceptable level for high throughput sequencing over a period of 4 months. By downloading sequences from GenBank and building partitions of new sequences on a weekly basis, an incrementally merged profile, consisting of an arbitrarily defined time period extending into the past, can be quickly generated. After the merged profile has been converted into a consensus sequence with an appropriate threshold set to achieve the desired sensitivity, the currently used primers can be tested *in silico *on the new consensus sequence to determine which regions will not have the minimum required coverage depth due to predicted primer failure. New primers can then be designed on these regions based on the new consensus. Because all of the sequences are included in the cumulative position profile, manual intervention is not necessary to decimate redundant sequences. As a result, it is possible to generate a consensus sequence that more closely represents the genotypes of the viral variants in circulation accurately and in a timely manner.

### Estimation of empirical sequencing quality values

**Figure 10:**
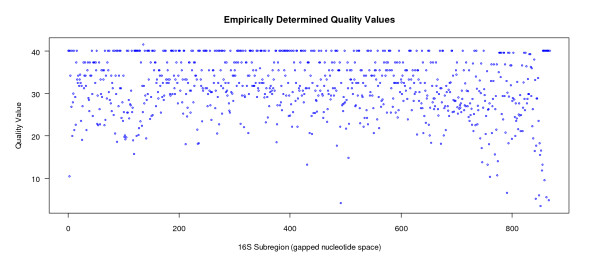
**Example plot of empirically determined quality values (QV)**. A subregion of the 16S gene was deeply sequenced with 454 Titanium and then transformed into a position profile. The average empirically determined QV was plotted on the y-axis, along the length of the genome on the x-axis.

To identify the nature of sequencing artifacts and how they may impact the analyses of a 16S metagenomic sample, ANDES was applied to compute empirically-determined quality values on a set of 454 Titanium sequencing runs. These actual quality values were compared against those assigned by the 454 base caller, or associated with sequence features, such as homopolymer runs. Briefly, a mock community consisting of 22 known microbial organisms from the phyla Actinobacteria, Bacteroidetes, Deinococcus-Thermus, Firmicutes, and Proteobacteria was established. PCR amplification targeted 3 variable regions of the ribosomal 16S gene, each approximately 500 bp in length. PCR products were then sequenced using 454 Titanium. The resultant sequences were then analyzed using the BLAST algorithm [15] against the 16S reference database consisting only of the mock community, in order to associate each sequence with an organism and 16S copy with the highest similarity. For each (organism, 16S copy, variable region) combination, a partition of sequences was created and AMOScmp was used to align the partitioned sequences against the specific (organism, 16S copy, variable region) reference sequence. A position profile was then generated based on the AMOScmp results. Figure 10 is an example of the estimated quality values along one of the (organism, 16S copy, variable region) combination partitions. The average coverage depth was 10,514. The majority of positions had error rates of less than 1 in 100 (QV > 20). These average QVs were then compared against the average of QVs for each position assigned by the 454 base caller in order to determine how well 454 assigned quality values correlated to actual error rates. Further correlation analyses were also performed after aggregating by nucleotide type and length of homopolymer runs (not shown).

The results from the studies indicate that there was little correlation between empirically determined quality values and the 454 assigned quality values. This suggests that many of the sequencing errors are unpredictable with current error models. The consensus sequences from 454 assemblies are generally of high quality because the availability of coverage depth ameliorates the impact of random errors. Systematic errors, such as those caused by homopolymer runs, which cannot be addressed with additional sequencing depth, are known and accepted to be a source of error that needs be addressed in downstream processing. However, the level of individual error rates cannot be dismissed if they are intended for downstream cluster analyses, an important step prior to establishing operational taxonomic units (OTUs). An example of the frequency of errors along the length of an (organism, 16S copy, variable region) combination is shown in Figure 10. This pattern of random error along the entire length of the alignment is consistent with the difficulties that have been observed when using individual 454 sequences to establish OTUs in order to estimate population diversity [16]. Contrary to initial supposition, addressing errors from homopolymer runs alone is not sufficient to solving the problem of accurately establishing OTUs. Tools such as ANDES, which allow an investigator to produce an accurate local and global view of deep sequencing results, serves to make the identification of these trends more visible, so they may eventually be addressed correctly.

### Future work

As discussed in the "Merging and comparing profiles" subsection, the alignment of two profiles uses the major allele for each positional distribution as the proxy for the alignment. While this works well for most datasets that have been analyzed, it would be preferable to use the RMSD distances between positions as actual mismatch penalties. This would make the full nucleotide distribution at each position available for optimally determining whether introducing a gap or a substitution was more suitable.

## Conclusions

The increasing cost efficiency of deep sequencing provides us with a great opportunity for asking questions about and exploring the potentially complex structure of variations within an evolving, nearly homogeneous population or a shared biomarker. We have explored the application of the ANDES tool sets toward understanding mutation rates of a coronavirus, aiding in the cluster analyses of seasonal versus swine origin H1N1 flu partitions, and exploring the quality of a deep sequencing technology itself. ANDES provides a useful tool set and a basis for the comparison of complex samples at the level of positional nucleotide distributions, both visually and statistically. These tools will help to reveal the important biological information hidden under mountains of deep sequencing data.

## Availability and requirements

**Project name: **ANDES

**Project home page: **http://andestools.sourceforge.net/

**Download web site: **https://sourceforge.net/projects/andestools

**Operating system: **Tested and in production on Linux.

**Programming language: **Perl and R

**License: **GNU GPL V3

**Any restrictions to use by non-academics: **none

## Competing interests

The authors declare that they have no competing interests.

## Authors' contributions

BAM, SY, MRD, LDE, DJS, TBS conceived of the study and participated in its design. KL, EV wrote the software. LDE, EV, KL, SY, MRD, BAM analyzed the data and evaluated results. BAM, MRD, LDE designed and ran experiments and generated data. KL wrote the manuscript. All authors read and approved the submitted manuscript.
